# Neurovascular injury associated non-apoptotic endothelial caspase-9 and astroglial caspase-9 mediate inflammation and contrast sensitivity decline

**DOI:** 10.1038/s41419-022-05387-3

**Published:** 2022-11-08

**Authors:** Crystal Colón Ortiz, Albertine M. Neal, Maria I. Avrutsky, Monica Choi, Jade Smart, Jacqueline Lawson, Carol M. Troy

**Affiliations:** 1 Department of Pathology & Cell Biology; Vagelos College of Physicians and Surgeons, Columbia University, New York, NY 10032 USA; 2 Barnard College, Columbia University, New York, NY 10032 USA; 3 Department of Neurology; Vagelos College of Physicians and Surgeons, Columbia University, New York, NY 10032 USA; 4 The Taub Institute for Research on Alzheimer’s Disease and the Aging Brain; Vagelos College of Physicians and Surgeons, Columbia University, New York, NY 10032 USA

**Keywords:** Neuro-vascular interactions, Cell signalling

## Abstract

Retinal neurovascular injuries are a leading cause of vision loss in young adults presenting unmet therapeutic needs. Neurovascular injuries damage homeostatic communication between endothelial, pericyte, glial, and neuronal cells through signaling pathways that remain to be established. To understand the mechanisms that contribute to neuronal death, we use a mouse model of retinal vein occlusion (RVO). Using this model, we previously discovered that after vascular damage, there was non-apoptotic activation of endothelial caspase-9 (EC Casp9); knock-out of EC Casp9 led to a decrease in retinal edema, capillary ischemia, and neuronal death. In this study, we aimed to explore the role of EC Casp9 in vision loss and inflammation. We found that EC Casp9 is implicated in contrast sensitivity decline, induction of inflammatory cytokines, and glial reactivity. One of the noted glial changes was increased levels of astroglial cl-caspase-6, which we found to be activated cell intrinsically by astroglial caspase-9 (Astro Casp9). Lastly, we discovered that Astro Casp9 contributes to capillary ischemia and contrast sensitivity decline after RVO (P-RVO). These findings reveal specific endothelial and astroglial non-apoptotic caspase-9 roles in inflammation and neurovascular injury respectively; and concomitant relevancy to contrast sensitivity decline.

## Introduction

Neurovascular hypoxic-ischemic injuries in the retina are a leading cause of degenerative vision loss [1] characterized by impairment of the retinal vasculature through aberrant neovascularization and decreased blood flow. Neurovascular injuries damage the communication between endothelial, pericytes, glial, and neuronal cells that form the neurovascular unit (NVU). Disruption of the NVU results in the breakdown of the blood-retinal barrier (BRB), edema, inflammation, retinal neuronal atrophy, and visual dysfunction [2]. While molecular signatures involved in retinal vascular pathology have been identified, the specific contribution of the injured endothelium and reactive gliosis to vision dysfunction and neuronal death remain to be established. Parsing out the roles of specific cell types in pathology associated with retinal neurovascular injuries can help identify new therapeutic targets.

Non-invasive in vivo visualization and manipulation of the NVU can be done in the retina. To study cell-specific roles of the components of the NVU in the context of neurovascular injury, we used a characterized mouse model of Retinal Vein Occlusion (RVO). RVO is the second most common cause of blindness after diabetic retinopathy amongst young adults [3] and current therapeutics do not prevent sustained chronic vision loss [2, 4, 5]. The laser-induced mouse model of RVO [6] is a noninvasive method and one of the best translational avenues to study the role of inflammation [7, 8] and identify signaling pathways involved in RVO pathology [9, 10]. Using the mouse model of RVO, we previously discovered that neurovascular injury induced non-apoptotic activation of endothelial caspase-9 (EC Casp9). Moreover, we found that EC Casp9 mediated retinal edema, capillary ischemia, and neuronal death [10], revealing a cell-specific role of caspase-9 in the pathophysiology of neurovascular injury.

Caspases are a group of cysteine-dependent aspartate-directed proteases that can activate cell death and inflammatory signaling. Over recent years their roles in the brain have been further characterized as relevant promoters of synaptic pruning, axon guidance, differentiation and in glial cells, as mediators of inflammation and filament aggregation [11]. Cell-specific nonapoptotic caspase expression in glial cells have been found in animal models of neurodegeneration and postmortem brain tissue. Evidence suggests that microglial caspase-8 is relevant for the release of pro-inflammatory cytokines [12] and is expressed in Parkinson’s and Alzheimer’s disease [12, 13]. Astroglial caspase-6 is a key element in Alexander’s disease [14] and known to mediate cleavage of the intermediate filament glial fibrillary acidic protein (GFAP) [15]. Our previous studies demonstrated that RVO induced high non-apoptotic levels of cl-caspase-6 in astrocytes, which were significantly downregulated by the administration of the specific caspase-9 inhibitor Pen1-XBIR3 [10]. Besides caspase-6 function in GFAP cleavage [15], caspase-6 also promotes inflammation [16] and neurodegeneration [17, 18].

Retinal resident glial cells (microglia, Müller glia, and astrocytes) provide neuronal and vascular support and participate in neuropathology [19–21]. During retinal vascular injury, glial cells undergo changes in morphology, become reactive, and release inflammatory cytokines that lead to neuronal dysfunction, recruitment of leukocytes, and breakdown of the BRB [20–23]. Understanding the active interaction and contribution of glial cells to the dysregulation of the neuronal micro-environment and vasculature requires the characterization of the response of glial cells, which will illuminate their potential role in mediating neurodegeneration in retinal vascular disease.

Many studies assessing vitreous samples of RVO patients have identified increased levels of pro-inflammatory cytokines and chemokines [24–28]; and similar results are observed in the mouse model of RVO [6, 8]. The presence of inflammatory mediators in patients with RVO is correlated with increased foveal thickness and hypoxic-ischemic injury [27], underlining the relevance of understanding the source of these cytokines in the injured retina. Current lines of treatment for RVO include anti-angiogenic and anti-inflammatory drugs. The first line of treatment targets vascular endothelial growth factor (VEGF), which acts on endothelial cells and promotes neovascularization that leads to edema [29]. Corticosteroid implants target inflammation and while they help to resolve macular edema they can also lead to cataract formation and increase of intraocular pressure [30]. Therefore, there is a need to better understand the inflammatory pathways in RVO to develop more specific therapeutics that simultaneously target neuroinflammation, neurodegeneration and retinal edema.

Here, we aimed to evaluate the contribution of endothelial and astroglial caspase-9 (Astro Casp9) to vision dysfunction, inflammation and RVO pathology. We found that deletion of EC Casp9 contributed to a decline in contrast sensitivity and modulated the levels of inflammatory cytokines and glial responses in a time-dependent manner in RVO. Additionally, we discovered that knock-out of Astro Casp9 reduced capillary ischemia and contrast sensitivity decline.

## Materials and methods

### Animal husbandry and care

All animals were handled in accordance with the Association for Research in Vision and Ophthalmology (ARVO) statement for the use of animals in ophthalmic and vision research and monitored by the Institutional Animal Care and Use Committee (IACUC) of Columbia University.

Animals used in this study were 2-month-old males and females. The tamoxifen-inducible EC Casp9 mouse line was generated by crossing Cdh5(Pac)-Cre/ERT2 [31] with caspase-9 flox/flox mice [10]. The EC Casp9 WT/KO line was validated and reported in [10] and the Cdh5(Pac)-Cre/ERT2 promoter has been shown previously to have strong specificity to endothelial cells [32]. The tamoxifen-inducible Astro Casp9 mouse line was generated by crossing the hGFAP-Cre/ERT2 to the B6.Cg-Tg(GFAP-Cre/ERT2) 505Fmv/J (Jackson Stock No: 012849 | GCE [33]). The GFAP promoter has been previously characterized [33] and used in a mouse model of glaucoma to knockout caspase-8 specifically in astrocytes [34].

**Fig. 1:**
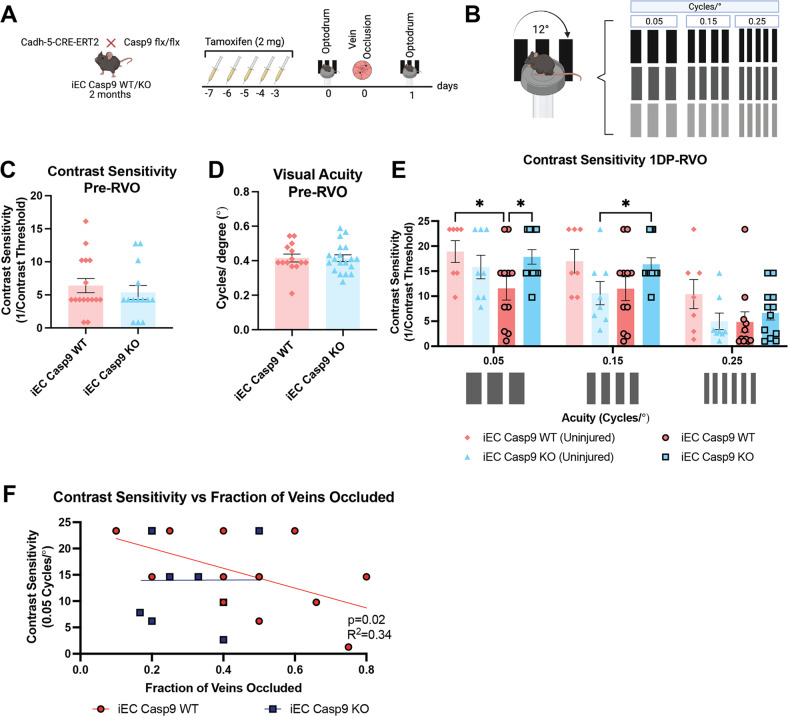
EC Casp9 deletion rescues contrast sensitivity one day P-RVO. **A** Experimental schematic. Two-month-old iEC Casp9 WT/KO mice were treated with tamoxifen for five consecutive days. After two days of rest, their optokinetic response was tested pre and one-day P-RVO. Created with BioRender.com. **B** Optokinetic test was performed by testing their contrast sensitivity at three acuity settings: 0.05, 0.15, 0.25 cycles/°. **C–D** Contrast sensitivity and visual acuity values prior to RVO of uninjured iEC Casp9 WT (*n* = 14–15) and KO (*n* = 14–19). **E** Contrast sensitivity response of uninjured iEC Casp9 WT (*n* = 7) and KO (*n* = 8), and iEC Casp9 WT (*n* = 11) and KO (*n* = 12) one-day P-RVO at 0.05, 0.15, 0.25 cycles/°. *P ≤ 0.05, Error bars mean ± SEM, Two-way ANOVA, Fisher’s LSD test. **F** Contrast sensitivity correlated with fractions of veins occluded, linear regression *n* = 12.

Cre recombination was induced in both colonies after five consecutive days of 2 mg tamoxifen intraperitoneal injections (100 μL of 20 mg/mL). Tamoxifen was dissolved in corn oil (20 mg/mL), then 2 mg of tamoxifen was administered intraperitoneally (IP) for five consecutive days when animals were eight weeks old (Fig. 1A).

### Mouse model of retinal vein occlusion (RVO)

RVO was induced in major retinal veins (*n* = 2–3 veins occluded/eye) ten minutes after tail vein injection of the photoactivatable dye, Rose Bengal (37.5 mg/kg). Eight minutes after injection, eyes were dilated with tropicamide, and phenylephrine chloride eye drops, and mice were anesthetized with IP administration of a cocktail of ketamine (80–100 mg/kg) and xylazine (5–10 mg/kg). Two minutes after injection, depth of anesthesia was confirmed by toe-pinch. Afterwards, retinal veins were irradiated with the Micron IV image guided laser (532 nm) from Phoenix Research labs by delivering three adjacent laser pulses (power 100 mW, spot size spot size 50 μM, duration 1 second, total energy 0.3 J) to each vein at a distance of 375 μM from the optic nerve head. RVO occlusions were examined by fundus imaging after irradiation and one day after RVO (P-RVO). Occluded veins were identified by a whitening of the vessel at the site of the occlusion, dilation distal to the occlusion, and constriction of vessel diameter proximal to the occlusion. Persistent occlusions from the time of irradiation up to one day P-RVO, were classified as successful occlusions. Retinas with detachment, subretinal hemorrhage, or full ischemia one day P-RVO were excluded from further analysis. A detailed description of the RVO mouse model can be found in [35].

### Image guided optical coherence tomography (OCT) and analysis

Following anesthesia and pupil dilation as indicated above in the mouse model of RVO section, OCT images were captured using the Phoenix Micron IV image-guided OCT system. Two vertical and two horizontal OCT scans were taken 75 µm from the periphery of the RVO burn areas, pre- RVO, and one-day P-RVO. Total retinal thickness was determined using the InSight software.

### Disorganization of inner retinal layers (DRIL)

Four OCT images per retina were analyzed in Image J for presence of DRIL. DRIL was measured with a horizontal line of indistinctive boundaries between the inner nuclear layer (INL) and the outer plexiform layer (OPL).

### Optokinetic response test

In vivo examination of the mouse optokinetic response was performed using the Striatech Optodrum. The awake mouse was placed on the platform inside the arena where the Optodrum presented a rotating stripe pattern to the animals at a rotation speed of 12°/second. Contrast sensitivity (1/contrast threshold) was determined by assessing the contrast threshold after presenting incremental visual acuity patterns at 0.05, 0.15 and 0.25 cycles/°. Readings were taken one-day pre-RVO, one and two days P-RVO.

### Immunohistochemistry (IHC) and imaging

Mice were perfused with sterile saline and then with 4% paraformaldehyde (PFA). Eyes were enucleated and immersed in 4% PFA overnight at 4 °C, they were then washed three times for 10 min with 1X PBS and immersed in 30% sucrose for three nights at 4 °C. Eyes were embedded in optimal cutting temperature solution and kept at −80 °C. Embedded eyes were sectioned at 20 µm thickness. Retinal slides were chosen to match approximate location of OCT scans and then were washed with 1X PBS for 5 min. Sections were permeabilized with 0.1% Triton X-100 for two hours and blocked with blocking buffer (10% Normal Goat Serum (NGS), 1% Bovine Serum Albumin (BSA) (dissolved in 1X PBS and filtered) overnight at 4 °C. Primary antibodies for Iba-1 (1:200, BioCare CP29013), CD68 (1:100 BioRad MCA1957GA), cl-caspase-6 (1:100, Cell Signaling 9761 S), GFAP (1:2,000 AVES GFAP), nestin (1:100, AVES Nes), and AQP-4 (1:100 Cell Signaling 59678 S) were prepared with blocking buffer and sections were incubated overnight at 4 °C. Afterwards, sections were washed four times with 1X PBS for 5 min, secondary antibodies were diluted in blocking buffer (1:1,000) and sections incubated for two hours at room temperature. Then, sections were washed four times with 1X PBS for five minutes and then stained with nuclear staining (Hoechst, 1:5,000 dilution) for 5 min. After one wash with 1X PBS for five minutes, retinal sections were mounted with Fluoromount-G. Retinas were imaged using a spinning disk confocal microscope (BioVision Technologies) in which z-stack images were taken.

### Deoxynucleotidyl transferase dUTP nick end labeling (TUNEL)

Sections were first permeabilized with 0.1% Triton X-100 for two hours. TUNEL was then done following the manufacturer’s protocol (Promega G3250). Sections were then processed for IHC as described above.

### Analysis of cell counts

Images were first processed using FIJI and brightness and contrast settings were adjusted to appropriate parameters based on uninjured (no RVO) or positive control (TUNEL) retinal tissue and background. Then, the number of cells were quantified based on colocalization with cellular marker or nuclei marker using the multi-point function in FIJI. Analysis was performed by an experimenter who was blinded to genotype and treatment. Values were averaged by images per section and then averaged values of sections per eye. More details about IHC quantification of cells can be found in [36].

### Analysis of percent area of expression

Images were first processed using FIJI by adjusting the scale first based on the magnification at which images were taken. Area of interest was selected with the polygon setting. Then, the thresholding setting was set up based on levels of expression of uninjured retinal tissue. The same thresholding parameter was then used to quantify the percent area of expression in all retinal images by an experimenter that was blinded to genotype and treatment. Values were averaged by images per section and then averaged values of sections per eye.

### Microglial morphology analysis

Images were processed using FIJI by adjusting the scale, then applying the thresholding setting that was based on uninjured retinal tissue. Afterwards, the analyze particle function was applied to identify particles bigger than 49 µm and measurements settings were adjusted to determine microglia area, shape description, integrated density, centroid, perimeter, and Ferret’s diameter. Values were averaged by images per section and then averaged values of sections per eye.

### Microglial area of colocalization

Images were processed using FIJI and after setting up the scale and selecting the brightness and contrast parameters of expression for Iba-1 and CD68, images were converted to RGB color. Then, color threshold function was applied and used to measure the area of colocalization. Values were averaged by images per section and then averaged values of sections per eye.

### Western blot and analysis

Animals were perfused with saline, and retinas were then dissected, minced, and placed in RIPA buffer with protease (Fisher) and phosphatase inhibitors II and III (Sigma). Tissue was then sonicated on ice with a Sonic Dismembrator (Model 500, Fisher Scientific) for one minute (1 second on/ 1 second off). Retinas were matched one-day P-RVO based on the fraction of veins occluded and disorganization of inner retinal layers (DRIL) percentages, two matched retinas were then combined. Protein concentrations were determined using a BCA assay. A total of 100 µg of retinal protein was loaded per sample. Total protein transfer was determined using REVERT (LI-COR). Protein blots were blocked with LI-COR Blocking Buffer for one hour at room temperature and then incubated for two nights with primary antibody (GFAP GA5 Sigma 63893) at 4 °C. Blots were washed three times with 0.1% TBS-Tween 20 and then incubated with secondary antibodies at 1:5,000 overnight at 4 °C. After three ten-minute washes with 0.1% TBS-Tween 20, blots were imaged using the LI-COR Odyssey Imaging System. Blots were analyzed using Image Studio Lite Software; all signals were first normalized to total protein loaded and then to the average of uninjured littermate control values.

### Cytokine arrays and analysis

Retinal tissue was prepared the same way as for Western Blot, except for the replacement of RIPA buffer with the manufacturer’s cell lysis buffer. Cytokine arrays were performed according to manufacturer’s protocol (Abcam, ab211069) and blots were imaged using the LI-COR Odyssey Imaging System and analyzed with Image Studio Lite Software. Analysis of the cytokine array was done following recommendation of the protocol (Abcam, ab211069). Averaged relative expression of each cytokine was normalized to each membrane’s positive control. Data is presented as relative expression levels.

### Statical analysis

The RVO model, in vivo and in vitro measures and analysis were done by investigators blinded to animal genotype. Eyes that presented with retinal detachment, cataract, excessive edema or intraretinal hemorrhage were excluded from all analyses and only eyes that had one or more occlusion by one day P-RVO were considered viable. GraphPad Prism and Excel were used for statistical analysis. One-way and Two-way ANOVA were used to determined statistical differences followed by Fisher’s LSD test; data are presented as mean ± SEM. Significance was set to be *p* < 0.05.

## Results

### EC Casp9 deletion rescues contrast sensitivity decline P-RVO

One of the effects of RVO is damage to visual function, including a significant decline in contrast sensitivity threshold [37, 38]. We sought to explore if our mouse model of RVO recapitulated visual dysfunction in the form of contrast sensitivity decline after injury. Since we previously found that EC Casp9 knockout resulted in decreased retinal edema and neuronal death in RVO [10], we also evaluated the role of EC Casp9 on vision function one-day P-RVO. To this end, we tested contrast sensitivity with incremental visual acuity frequencies of 0.05, 0.15, and 0.25 cycles/° (Fig. 1A, B) using the automated optomotor reflex test; OptoDrum [39]. First, we probed if there were any differences in visual function between uninjured iEC Casp9 WT and iEC Casp9 KO mice by performing separate contrast sensitivity and visual acuity tests (Fig. 1C, D). The results indicate that there were not any differences in visual function performance between genotypes. We then proceeded to test contrast sensitivity discrimination with the incremental visual acuity frequencies and found that the mouse model of RVO caused a significant decrease in contrast sensitivity at 0.05 cycles/°. Moreover, the deletion of EC Casp9 rescued the observed contrast sensitivity decline one-day P-RVO (Fig. 1E). No differences between the groups were found when assessing contrast sensitivity at higher acuity frequencies. We then evaluated if the decline in contrast sensitivity was associated with the fractions of veins occluded, as we have previously found that the fractions of veins occluded has a positive correlation with the percentage of retinal thickness or edema [10]. We found that the degree of contrast sensitivity declines significantly correlated with the fractions of veins occluded in iEC Casp9 WT but not in iEC Casp9 KO animals (Fig. 1F), indicating that as a result of the occlusion EC Casp9 contributes to contrast sensitivity decline.

### EC Casp9 regulates an increase in inflammatory cytokines one day P-RVO

**Fig. 2:**
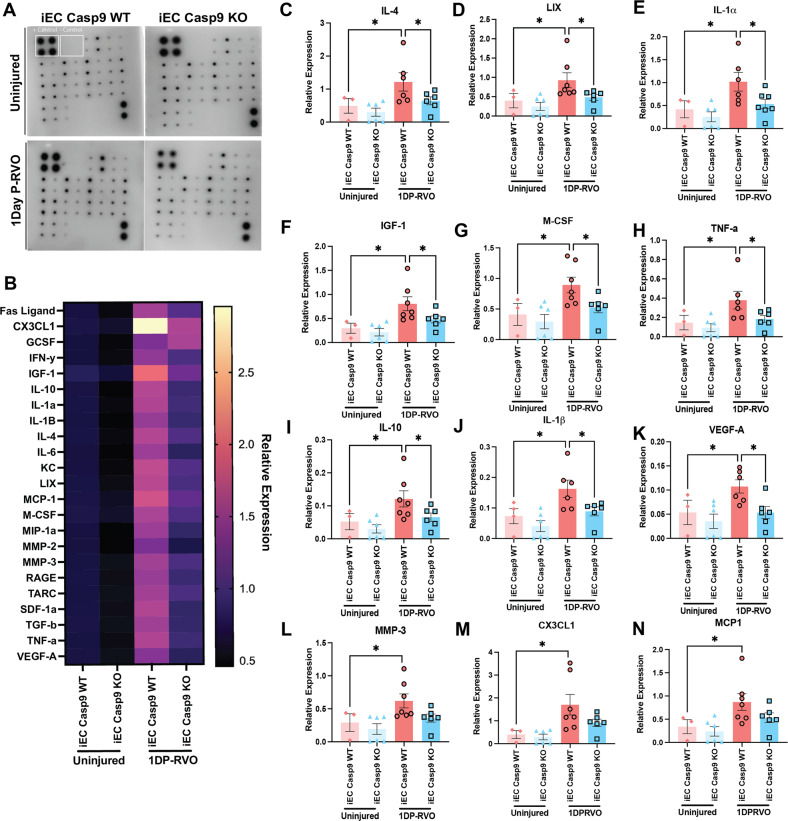
EC Casp9 regulates an increase in inflammatory cytokines one day P-RVO. **A** Representative images of cytokine arrays of retinas from uninjured iEC Casp9 WT/KO and one day P-RVO iEC Casp9 WT/KO mice. **B** Heat map array of relative expression of 23 cytokines normalized to positive signal per array blot of iEC Casp9 WT in uninjured iEC Casp9 WT (*n* = 3) and KO (*n* = 6) and iEC Casp9 WT (*n* = 6) and KO (*n* = 6) one day P-RVO. **C**–**N** Relative expression of induced cytokines one day P-RVO. Error bars mean ± SEM, One-way ANOVA, Fisher’s LSD test. *P ≤ 0.05.

After we found that EC Casp9 promoted a decline in vision function, we sought to evaluate the EC Casp9 downstream signaling pathway which mediates loss of vision. Inflammatory cytokines are known to be a main contributor of neovascularization, and correlate with hypoxic-ischemic injury and retinal edema [27], and are implicated in RVO pathology (summarized in the recent review [40]). Our previous work revealed that the peak of retinal edema in the EC Casp9 mouse line is two days P-RVO which coincided with a significant decrease in hyperreflective foci (HRF) (a clinical biomarker of inflammation [41–43]) [10]. Thus, we proceeded to collect retinas one day P-RVO to perform a cytokine array and determine which inflammatory cytokines were present before the peak of edema and associated with EC Casp9 signaling. We found that RVO led to a significant increase of the following cytokines in the WT retinas: insulin growth factor-1 (IGF-1), IL-4, LIX (CXCL5), IL-1α, macrophage colony-stimulating factor (M-CSF), tumor necrosis factor (TNF-α), IL-1β, IL-10, and vascular endothelial growth factor A (VEGF-A); the cytokine changes were attenuated in injured EC Casp9 KO retinas (Fig. 2A–L). Other cytokines that we found to be upregulated in RVO independently of EC Casp9 were CX3CL1 (fractalkine), MMP3 and MCP1 (Fig. 2M, N). We also found that MMP-2, SDF-1α, and TGF-β were decreased in injured iEC Casp9 KO compared to injured WT, but these cytokines were not significantly modulated by RVO in iEC Casp9 WT. (Fig. S1A–C).

We then assessed potential protein-protein interactions of caspase-9 with RVO-induced and EC Casp9-regulated cytokines by performing STRING [44]. The STRING database predicts physical and functional protein-protein interactions based on primary literature textmining, annotated pathways tested experimentally, computational predictions based on conserved genomics, and organism-to-organism interaction evidence [45]. The STRING analysis suggests potentially significant caspase-9 interactions, as experimentally determined, with the TNF-superfamily and IL-1β (Fig. S2A, pink lines). Other potential caspase-9 interactions with IL-10, IGF-1, and VEGF-A are suggested through textmining (Fig. S2A, green lines). Similar levels of significant interactions were found when we evaluated the caspase-9 downstream targets, caspase-7 and caspase-6 using STRING analysis (Fig. S2B, C). The STRING analysis suggests that caspase-9 and downstream executioner caspases could potentially interact with cytokines to be processed or released in a soluble form as caspases are known to be relevant for cytokine processing [46–48].

### EC Casp9 deletion modulates microglial CD68 in a time-dependent manner P-RVO

**Fig. 3:**
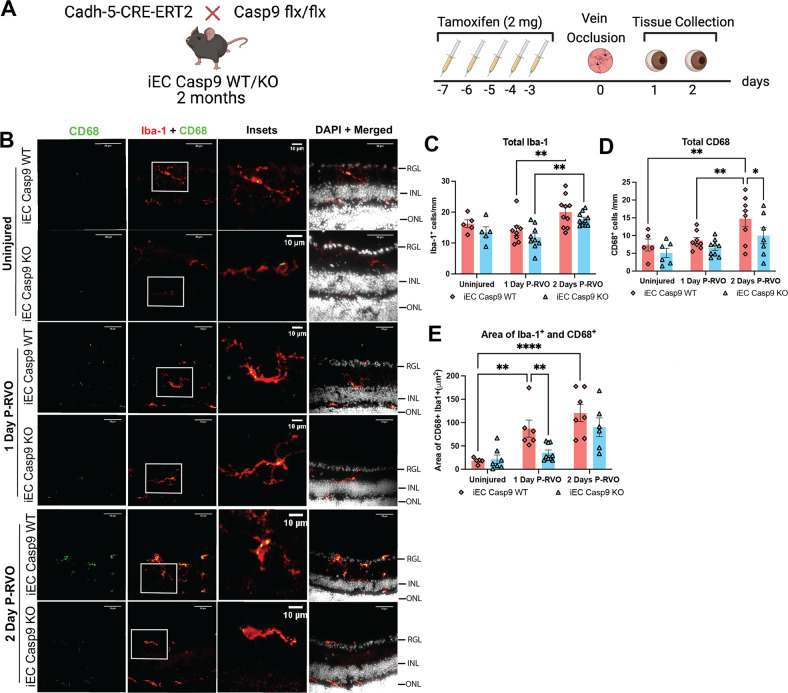
RVO induces an EC Casp9 regulated increase in CD68^+^ microglia. **A** Experimental schematic. Two-month-old iEC Casp9 WT/KO mice were treated with tamoxifen for five consecutive days. After two days, eyes were subjected to RVO, and eyes were collected one and two days P-RVO. Created with BioRender.com. **B** Retinal cross-sections from uninjured, one and two days P-RVO iEC Casp9 WT and KO mice were stained with CD68 (green), Iba-1 (red), and DAPI (white). Scale bar 50 µm and inset 10 µm. **C** Quantification of the number of Iba-1^+^ cells in uninjured iEC Casp9 WT (*n* = 5) and KO (*n* = 6), one day P-RVO iEC Casp9 WT (*n* = 8) and KO (*n* = 9), and two days P-RVO iEC Casp9 WT (*n* = 10) and KO (*n* = 10 **D** Quantification of number of CD68^+^ cells in uninjured iEC Casp9 WT (*n* = 5) and KO (*n* = 6), one day P-RVO iEC Casp9 WT (*n* = 8) and KO (*n* = 9), and two days P-RVO iEC Casp9 WT (*n* = 8) and KO (*n* = 7). **E** Quantification of percent area of colocalization of Iba-1^+^ and CD68^+^ cells in uninjured iEC Casp9 WT (*n* = 5) and KO (*n* = 5), one day P-RVO iEC Casp9 WT (*n* = 6) and KO (*n* = 9), and two days P-RVO iEC Casp9 WT (*n* = 7) and KO (*n* = 6). *P ≤ 0.05, **P ≤ 0.01, and ****P ≤ 0.0001. Error bars mean ± SEM, Two-way ANOVA, Fisher’s LSD test. Retinal ganglion layer (RGL), inner nuclear layer (INL), and outer nuclear layer (ONL).

Microglia are recognized as the immune cells of the CNS, and in RVO have been identified to be a major contributor of pro-inflammatory cytokines and linked to retinal ganglion cell (RGC) death [7, 8]. In the context of neurovascular injury microglia undergo morphological and molecular inflammatory changes [49]. Therefore, we sought to investigate whether EC Casp9 regulates pro-inflammatory microglial responses (Fig. 3A). We assessed the total number of microglia by immunohistochemistry (IHC) with the microglial marker, Iba-1, of uninjured, one and two days P-RVO retinal sections (Fig. 3B). Quantification analysis showed that the total number of Iba-1^+^ cells increase significantly two days P-RVO when comparing one-day P-RVO to two-days P-RVO retinas, independent of EC Casp9 expression (Fig. 3C). We then evaluated the expression of the microglial lysosomal marker, CD68, that is associated with a microglial inflammatory state and is increased in RVO [7, 50], by quantifying the total number of CD68^+^ cells. The data indicate that RVO induces a significant increase in the number of CD68^+^ cells by two days P-RVO compared to uninjured and one-day P-RVO iEC Casp9 WT retinas. Knockout of EC Casp9 led to a significant decrease in the total number of CD68^+^ cells compared to injured iEC Casp9 WT retinas two days P-RVO (Fig. 3D). To further determine the level of CD68 in microglial cells, we analyzed the area of colocalization between Iba-1 and CD68. The results demonstrate that RVO induces a significant upregulation of CD68 in microglia one and two days P-RVO, compared to uninjured iEC Casp9 WT retinas. EC Casp9 deletion led to significantly less induction of CD68 in Iba-1 cells one-day P-RVO, a trend that is sustained through two days P-RVO (Fig. 3E). Furthermore, we assessed whether RVO modulated microglial morphology in the context of EC Casp9. Our analysis indicates that microglia become bigger, hypertrophic, and more circular one-day P-RVO compared to uninjured iEC Casp9 WT retinas, and these changes in area and Ferret’s diameter are blocked by deletion of EC Casp9 one day P-RVO, but by two days P-RVO there is no significant difference between genotypes (Fig. S3A–C). At two days P-RVO the change in microglia circularity decreases in both genotypes (Fig. S3D). Taken together, these results indicate that microglia morphological states are dynamic after RVO.

### EC Casp9 deletion leads to macroglial changes in nestin and AQP-4 P-RVO in a time-dependent manner

**Fig. 4:**
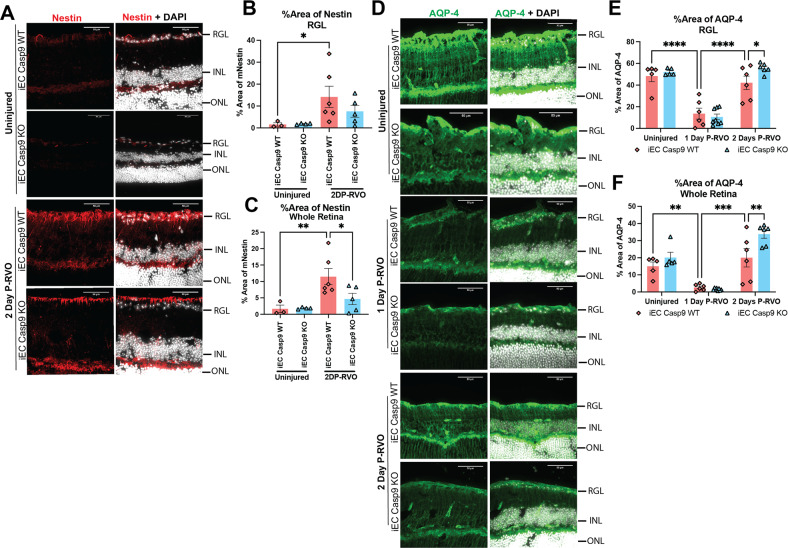
EC Casp9 induces macroglial changes in nestin and AQP-4 two days P-RVO. **A** Retinal cross-sections from uninjured and two days P-RVO iEC Casp9 WT and KO mice were stained with nestin (red) and DAPI (white). Scale bar=50 µm. **B** Quantification of percent area of expression of nestin in RGL and NFL of uninjured iEC Casp9 WT (*n* = 3) and KO (*n* = 5) and two days P-RVO iEC Casp9 WT (*n* = 6) and KO (*n* = 5). **C** Quantification of percent area of expression of nestin in whole retina of uninjured iEC Casp9 WT (*n* = 3) and KO (*n* = 5) and two days P-RVO iEC Casp9 WT (*n* = 6) and KO (*n* = 5). Error bars mean ± SEM, One-way ANOVA, Fisher’s LSD test. **D** Retinal cross-sections of uninjured, one and two-days P-RVO iEC Casp9 WT and KO stained with AQP-4 (green) and DAPI (white). Scale bar=50 µm. **E** Quantification of percent area of expression of AQP-4 in RGL and NFL of uninjured iEC Casp9 WT (*n* = 5) and KO *(n* = 5) one day P-RVO iEC Casp9 WT (*n* = 6) and KO (n = 8), and two days P-RVO iEC Casp9 WT (*n* = 6) and KO (*n* = 6). **F** Quantification of percent area of expression of AQP-4 in whole retina of uninjured iEC Casp9 WT (*n* = 6) and KO (*n* = 8) one day P-RVO iEC Casp9 WT (*n* = 10) and KO (*n* = 6), and two days P-RVO iEC Casp9 WT (*n* = 6) and KO (*n* = 8). Error bars mean ± SEM, Two-way ANOVA, Fisher’s LSD test. *P ≤ 0.05, **P ≤ 0.01, ***P ≤ 0.001 and ****P ≤ 0.0001. Retinal ganglion layer (RGL), inner nuclear layer (INL), and outer nuclear layer (ONL).

Because other retinal glial cells, Müller glia and astrocytes, are also involved in the release of inflammatory cytokines, we looked at markers of macrogliosis including nestin and aquaporin-4 (AQP-4). Nestin is an intermediate filament whose expression is downregulated in mature macroglia [51], but in hypoxic-ischemic injury, nestin increases and leads to changes in macroglial motility and cell division [52, 53]. AQP-4 regulates a water channel protein whose low levels are implicated in retinal vascular disease. Knockdown of AQP-4 in a model of diabetic retinopathy resulted in increased expression of inflammatory molecules, including IL-6 and VEGF [54]. Moreover when AQP-4 was knocked-out there was BRB impairment [55] and in a model of neuromyelitis optica, AQP-4 loss led to increased retinal thickness [56]. To determine if RVO and EC Casp9 modulated these markers of macrogliosis, we assessed the levels of nestin and AQP-4 by IHC analysis and quantification of the percent area of expression in the RGL and in the whole retina. Data analyses show that RVO induces a significant increase in the level of nestin in the RGL and whole retina two days P-RVO (Fig. 4A–C), and that EC Casp9 deletion abrogates the increase in whole retina but does not alter the increase in RGL (Fig. 4A–C). Analysis of AQP-4 levels in the RGL and whole retina demonstrates that RVO induces a significant decrease in AQP-4 by one day P-RVO and that its levels are restored back to uninjured levels by two days P-RVO in the injured iEC Casp9 WT. Levels of AQP-4 in the iEC Casp9 KO were significantly higher than in the iEC Casp9 WT two days P-RVO (Fig. 4D–F). We found that macroglial response to RVO is also dynamic and that deletion of EC Casp9 rescues inflammatory markers at two days P-RVO.

Next, we sought to determine if RVO and EC Casp9 induced changes in GFAP, as increased levels of GFAP are often associated with an inflammatory response of Müller glia and astrocytes [57]. IHC analysis of GFAP in the RGL reveals that there are no changes in the level of GFAP at early time points in RVO nor are GFAP levels modified by deletion of EC Casp9 (Fig. S4A, B). However, analysis of GFAP levels from the outer nuclear layer (ONL) to the inner plexiform layer (IPL), showed a significant increase of GFAP in injured iEC Casp9 WT one day P-RVO that is modulated by deletion of EC Casp9. No differences were noted in the expression of GFAP from ONL to IPL by two days P-RVO (Fig. S4-C). These results suggest that the increase of GFAP by one day P-RVO occurs in Müller glia.

### EC Casp9 deletion abrogates the RVO-induced increases in astroglial cl-caspase-6 and contributes to caspase-6-induced GFAP cleavage P-RVO

Prior work showed that pharmacological inhibition of caspase-9 decreased the levels of its downstream target caspase-6 in astrocytes P-RVO [10]. Ablation of caspase-6 is correlated with low levels of the pro-inflammatory cytokines TNF-α and IL-6 [58] and astroglial cl-caspase-6 has been found in brain samples of patients with Alzheimer’s [59] and Alexander’s disease [14].

**Fig. 5:**
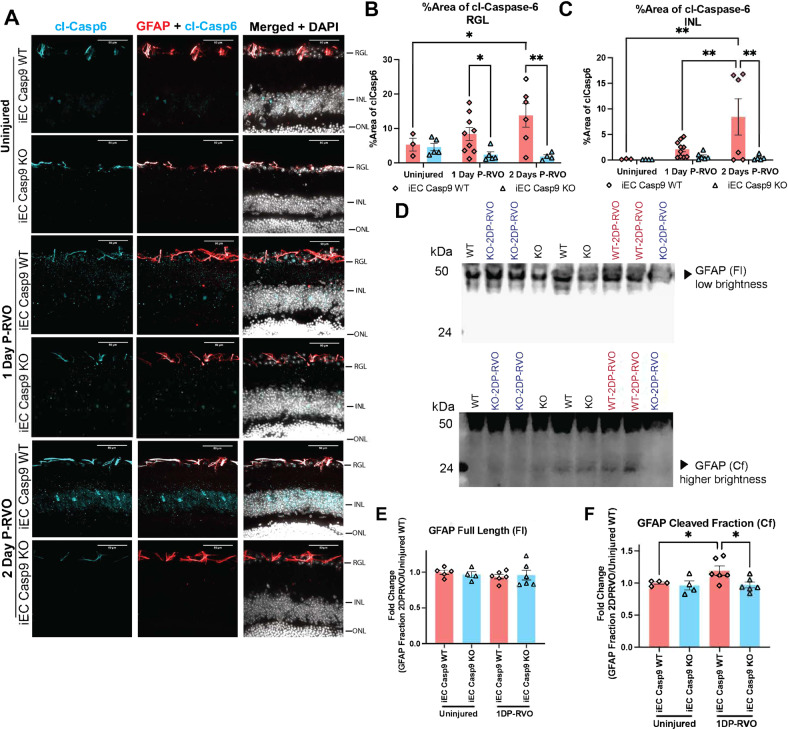
EC Casp9 deletion promotes astroglial decrease in cl-caspase-6 levels and caspase-6 GFAP cleavage P-RVO. **A** Retinal cross-sections from one and two days P-RVO iEC Casp9 WT and KO mice were stained with cl-caspase-6 (cyan), GFAP (red), co-localization of cl-Casp6 and GFAP (white), and DAPI (white). Scale bar=50 µm. **B** Percent area of expression of cl-caspase-6 in RGL and NFL of uninjured iEC Casp9 WT (*n* = 3) and KO (*n* = 5), one day P-RVO iEC Casp9 WT (*n* = 9) and KO (*n* = 6), and two days P-RVO iEC Casp9 WT (*n* = 6) and KO (*n* = 4). **C** Percent area of expression of cl-caspase-6 in INL of uninjured iEC Casp9 WT (*n* = 3) and KO (*n* = 5), one day P-RVO iEC Casp9 WT (*n* = 10) and KO (*n* = 6), and two days P-RVO iEC Casp9 WT (*n* = 6) and KO (*n* = 5). **D** Percent area of expression of GFAP in RGL and NFL of uninjured iEC Casp9 WT (*n* = 3) and KO (*n* = 4), one day P-RVO iEC Casp9 WT (*n* = 9) and KO (*n* = 5), and two days P-RVO iEC Casp9 WT (*n* = 4) and KO (*n* = 6). **E** Percent area of expression of GFAP from ONL to IPL in iEC Casp9 WT (*n* = 3) and KO (*n* = 4), one day P-RVO iEC Casp9 WT (*n* = 10) and KO (*n* = 6), and two days P-RVO iEC Casp9 WT (*n* = 4) and KO (*n* = 5). Error bars mean ± SEM, Two-way ANOVA, Fisher’s LSD test. Retinal ganglion layer (RGL), inner nuclear layer (INL), and outer nuclear layer (ONL). **F** Western blot detection of GFAP in uninjured iEC Casp9 WT and KO and two days P-RVO iEC Casp9 WT and KO retinal lysates. **G** Quantification of GFAP full length 50 kDa band in uninjured iEC Casp9 WT (*n* = 5, biological replicates) and KO (*n* = 4, biological replicates) and two-days P-RVO WT (*n* = 6, biological replicates) and KO (*n* = 6, biological replicates) normalized to total protein and average of uninjured iEC Casp9 WT. **H** Quantification of GFAP cleaved fraction 24 kDa in uninjured iEC Casp9 WT (*n* = 5, biological replicates) and KO (*n* = 4, biological replicates) and two-days P-RVO WT and KO normalized to total protein and average of uninjured iEC Casp9 WT. Data points present individual values. Error bars mean ± SEM, One-way ANOVA, Fisher’s LSD test. *P ≤ 0.05, **P ≤ 0.01.

To evaluate if EC Casp9 signaling was linked to cl-caspase-6 in astrocytes, we stained uninjured, one and two days P-RVO sectioned retinas from iEC Casp9 KO and WT littermates and then evaluated the level of cl-caspase-6 by quantifying the percent area of expression in the RGL, where astrocytes are located in the retina. The analysis revealed that RVO induces an increase in cl-caspase-6 two days after injury and that EC Casp9 deletion significantly blocks the change in cl-caspase-6 levels in the RGL one and two days P-RVO (Fig. 5A, B). Similar trends were also noted in the analysis of the level of cl-caspase-6 in the inner nuclear layer (INL), indicating an increase of cl-caspase-6 in the retinal neurons, which is blocked by EC Casp9 deletion two days P-RVO (Fig. 5C). We previously found that neuronal cl-caspase-6 led to neurodegeneration in a stroke model [17] and in RGC in a model of optic nerve crush [60].

To test the potential role of cl-caspase-6 in astrocytes, we assessed the only currently known function of astroglial cl-caspase-6; cleavage of GFAP [14, 59, 61]. To test if this was the case in RVO, we performed a western blot analysis of retinas two days P-RVO. For each sample, two retinas were matched by similar fractions of veins occluded and percentage of disorganization of inner retinal layers (DRIL), an indicator of capillary ischemia. Quantification of the GFAP 50 kDa band demonstrated that there were no differences in injured samples compared to uninjured regardless of genotype (Fig. 5F, G). However, analysis of the GFAP caspase-6 cleaved fraction, the 24 kDa band, showed that RVO induced a significant increase in GFAP cleavage in EC Casp9 WT compared to uninjured. This increase in GFAP cleavage was reduced to baseline levels in injured EC Casp9 KO (Fig. 5F, H). These data indicate that EC Casp9 induces cl-caspase-6 and GFAP cleavage in RVO.

### Astro Casp9 deletion decreased levels of astroglial cl-caspase-6 one day P-RVO

Our current and previous results suggest that caspase-9 is upstream of the activation of astroglial caspase-6. To evaluate whether Astro Casp9 is activating astroglial caspase-6 in RVO and neurodegeneration, we generated a tamoxifen-inducible Astro Casp9 knockout by crossing a caspase-9 flox/flox mice with a characterized astroglial promoter hGFAP-Cre ERT2 [33].

**Fig. 6:**
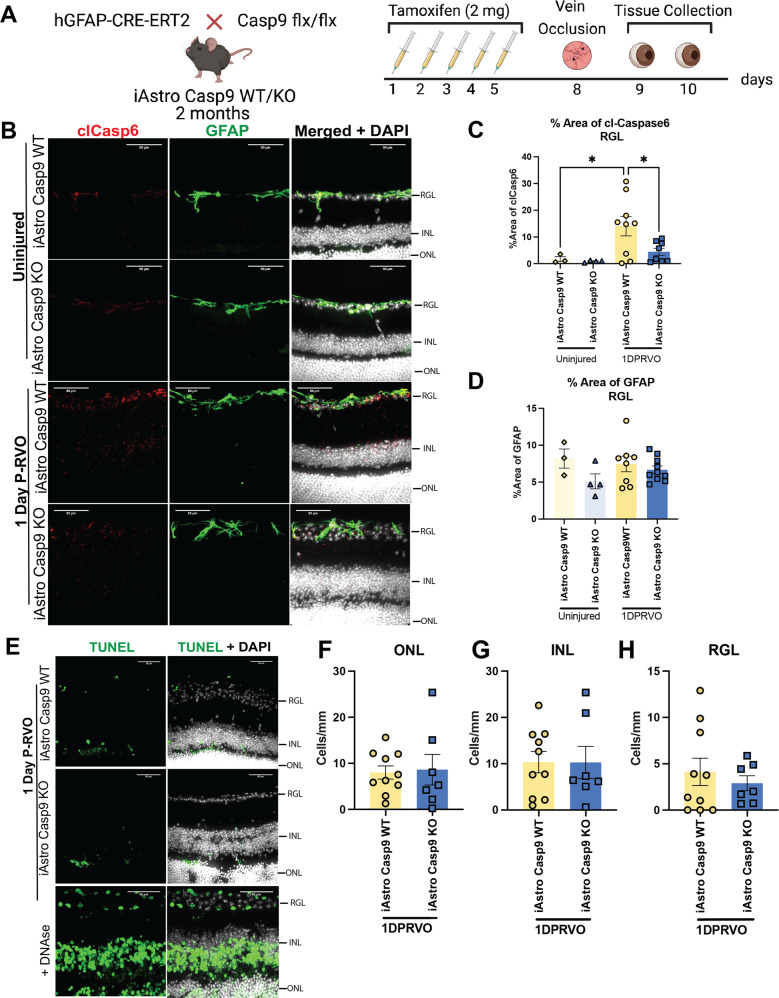
Astro Casp9 is upstream of astroglial cl-caspase-6. **A** Experimental schematic. Two-month-old iAstro Casp9 WT/KO mice were treated with tamoxifen for five consecutive days. After two days, animals were subjected to RVO, and eyes were collected one day P-RVO. Created with Biorender.com. **B** Retinal cross-sections from uninjured and one day P-RVO iAstro Casp9 WT and KO mice were stained with cl-caspase-6 (red), GFAP (green), and DAPI (white). **C** Percent area of cl-caspase-6 in RGL. Scale bar 50 µm. **D** Percent area of GFAP in RGL and NFL. **E** Retinal cross-sections from uninjured and one day P-RVO iAstro Casp9 WT and KO mice were stained with TUNEL (green) and DAPI (white). Number of cells that are TUNEL positive for **F** ONL, **G** INL, and **H** RGL. Error bars mean ± SEM, One-way ANOVA, Fisher’s LSD test. *P ≤ 0.05 and **P ≤ 0.01. Retinal ganglion layer (RGL), inner nuclear layer (INL), and outer nuclear layer (ONL). Scale bar = 50 µm.

We first assessed the levels of astroglial cl-caspase-6 P-RVO by performing IHC staining of cl-caspase-6 and quantifying the levels measuring the percent area of expression in uninjured and injured iAstro Casp9 WT and KO (Fig. 6A). The analysis revealed that RVO induced a significant increase in the levels of astroglial cl-caspase-6 in the RGL, which was downregulated in injured Astro Casp9 KO (Fig. 6B, C). Next, we assessed if Astro Casp9 was inducing any changes in astroglial GFAP. The data revealed that there are no differences in the levels of GFAP in the RGL regardless of genotype and injury (Fig. 6D). These results indicate that Astro Casp9 is upstream of astroglial cl-caspase-6 and is not implicated in changes of the intermediate filament GFAP at one day P-RVO.

To further investigate if Astro Casp9 was implicated in neuronal death P-RVO, we performed a deoxynucleotidyl transferase dUTP nick end labeling (TUNEL) which allows for immunodetection of cells with DNA degradation. Quantification of TUNEL^+^ nuclei of the different retinal layers suggest that there are not any significant differences between injured iAstro Casp9 WT and iAstro Casp9 KO (Fig. 6E–H). These data shows that Astro Casp9 and thus astroglial cl-caspase-6 are not relevant for neuronal death at one day P-RVO.

### Astro Casp9 deletion reduces capillary ischemia and contrast sensitivity decline one day P-RVO

**Fig. 7:**
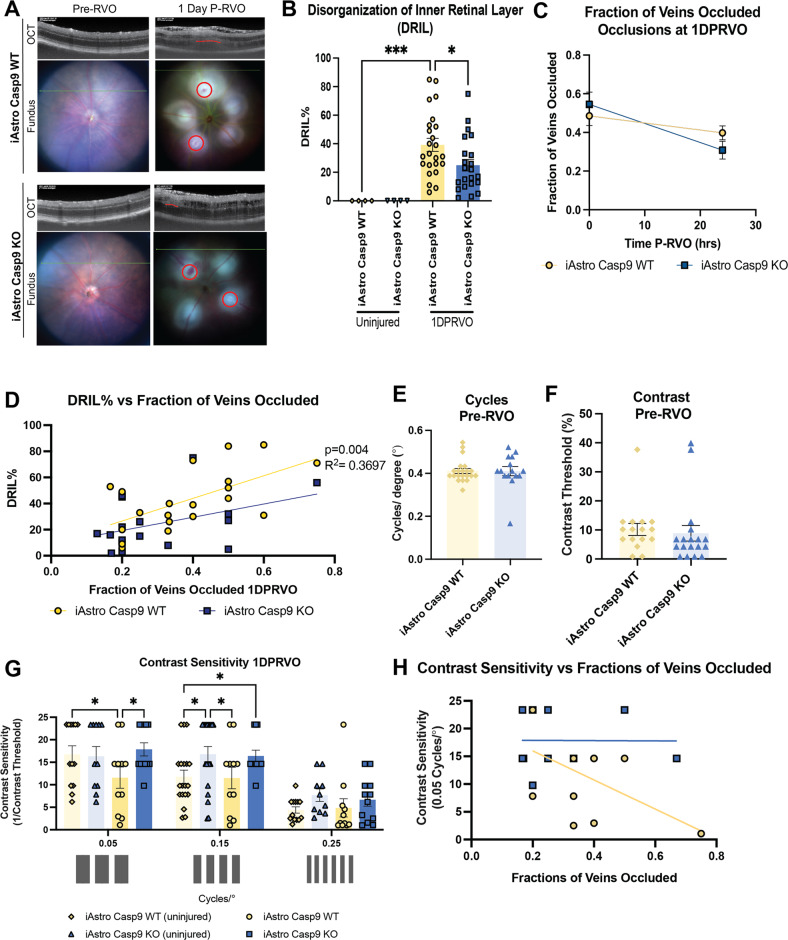
Astro Casp9 deletion reduces capillary ischemia and contrast sensitivity decline. **A** Representative retinal fundus and OCT images of DRIL of uninjured and one day P-RVO iAstro Casp9 WT and KO. Red strikes present DRIL areas. **B** Percentage of DRIL of uninjured and one day P-RVO iAstro Casp9 WT and KO. **C** Simple linear regression of DRIL vs fractions of veins occluded. **D** Timeline of fractions of veins occluded of iAstro Casp9 WT and KO. Astro Casp9 deletion rescues contrast sensitivity one day P-RVO. **E**–**F** Contrast sensitivity and visual activity values prior to RVO of uninjured iAstro Casp9 WT (*n* = 21) and KO (*n* = 15). **G** Contrast sensitivity response of uninjured iAstro Casp9 WT (*n* = 19) and KO (*n* = 20), and iAstro Casp9 WT (*n* = 16) and KO (*n* = 16) one day P-RVO at 0.0, 0.15, 0.25 25 cycles/°. Error bars mean ± SEM, Two-way ANOVA, Fisher’s LSD test. **H** Simple linear regression of contrast sensitivity at 0.05 cycles/° vs fraction of veins occluded at one day P-RVO. *P ≤ 0.05 and ***P ≤ 0.001.

Measurement of the disorganization of the inner retinal layers (DRIL) is used as a marker of capillary non-perfusion and correlates with visual acuity decline in RVO [62, 63]. We previously found that inhibition of caspase-9 or deletion of EC Casp9 significantly decreased DRIL levels [10]. To assess if Astro Casp9 contributed to capillary ischemia in RVO, we quantified the percent of DRIL in the retina one day P-RVO. The analysis suggests that RVO induced a significant increase in the area of DRIL in iAstro Casp9 WT compared to uninjured retinas. Moreover, the deletion of Astro Casp9 significantly ameliorated this increase (Fig. 7A, B). The fractions of veins occluded was the same for both genotypes (Fig. 7C), but the percent of DRIL positively correlated with the fractions of veins occluded in iAstro Casp9 WT, but not iAstro Casp9 KO (Fig. 7D). These data indicate that capillary ischemia could be driven by the fractions of veins occluded and Astro Casp9.

We previously found that inhibition of caspase-9 or ablation of EC Casp9 protected from retinal edema at early points P-RVO [10]. To evaluate the role of Astro Casp9 in retinal edema, we performed OCT before RVO and one day P-RVO and quantified the thickness of the retinal layers (Fig. S5). Analysis of the retinal thickness of iAstro Casp9 WT and KO in all layers indicate that one day P-RVO retinal layers become significantly thicker compared to uninjured iAstro Casp9 WT and KO. This finding implies that Astro Casp9 deletion does not protect from the development of retinal edema at one day P-RVO (Fig. S5**)**.

To investigate the role of Astro Casp9 in vision function we evaluated visual acuity and contrast sensitivity in uninjured iAstro Casp9 WT and KO which revealed that there were no significant differences between genotypes (Fig. 7E, F). Injured iAstro Casp9 WT mice presented a significant decline in contrast sensitivity at 0.05 cycles/°one day P-RVO when compared to uninjured iAstro Casp9 WT. This decline was rescued in animals with deleted Astro Casp9 (Fig. 7G). At 0.15 cycles/°, uninjured iAstro Casp9 KO had better contrast sensitivity compared to uninjured iAstro Casp9 WT. However, no differences were observed between injured iAstro Casp9 WT and KO at 0.15 cycles/°. Correlation analysis of contrast sensitivity vs fractions of veins occluded shows that there is a negative trend in injured iAstro Casp9 WT suggesting that the higher the fractions of veins occluded, the lower the contrast sensitivity. On the other hand, injured iAstro Casp9 KO present a flat correlation line regardless of fraction of veins occluded (Fig. 7H). These data indicate that Astro Casp9 is relevant for contrast sensitivity decline.

## Discussion

We have previously shown that non-apoptotic EC Casp9 regulates many of the retinal changes linked to retinal vascular injury, and now queried whether EC Casp9 was also required for vision impairment and inflammation. In this study, we showed that deleting caspase-9 in hypoxic endothelial cells regulates changes in visual function and inflammation in glial cells and cytokines. By using an established mouse model of RVO, we found that deleting EC Casp9 causes a decrease in: (1) contrast sensitivity decline, (2) inflammatory cytokines, and (3) glial cell reactivity. One of the observed EC Casp9 glial changes was increased levels of astroglial cl-caspase-6. We then were able to establish that Astro Casp9 was upstream of astroglial cl-caspase-6 and when Astro Casp9 was knocked-out, there was decreased capillary ischemia and contrast sensitivity decline P-RVO (Fig. S6).

RVO impacts contrast sensitivity, which has prospective use as indicator of disease progression [38, 64]. Contrast sensitivity measurements allow the detection of changes in retinal blood flow [64] in retinal vascular disease, serving as a good tool for diagnosing early disease pathology and treatment efficacy; and is also a reflection of RGC functionality as RGC are part of the contrast sensitivity pathway [65]. We found that RVO-induced contrast sensitivity decline was rescued one-day P-RVO when EC Casp9 or Astro Casp9 were deleted. The EC Casp9 contribution to contrast sensitivity decline is consistent with the role of EC Casp9 in mediating retinal edema and neuronal death [10]. Similarly, rescue of contrast sensitivity loss in Astro Casp9 genetic knock-out suggests that capillary ischemia contributes to vision dysfunction. Understanding the timing of vision contrast decline P-RVO and the role of EC Casp9 and Astro Casp9 in this process can help elucidate mechanisms of neurodegeneration and serve as potential biomarkers for retinal vascular disease. The observed contrast sensitivity decline could be due to degeneration and death of RGC types from the magnocellular or parvocellular vision pathways which are vulnerable and degenerate in RVO [66]. Contrast sensitivity decline can also occur preceding neuronal degeneration and be indicative of RGC synaptic dysfunction, which could occur due to the lack of proper oxygenation and nutrients for neurons, which are impaired by capillary ischemia and an inflammatory environment.

Pro-inflammatory cytokines are known to play an important role in RVO as they correlate with macular edema and hypoxic-ischemic injury [27]. Here, we found that deleting EC Casp9 regulated several pro-inflammatory cytokines (IGF-1, IL-1α, IL-1β, M-CSF, TNF-α, and VEGF-A), anti-inflammatory cytokines (IL-4 and IL-10), and a chemokine (LIX). A study that evaluated the levels of cytokines and chemokines in vitreous samples from human patients with ischemic RVO showed increased levels of TNF-α, IL-1β, IL-4, and IL-10 [67], which we found to be decreased by EC Casp9 deletion. This evidence highlights the translational relevance of our model system. The importance of a more detailed understanding of the inflammatory pathways initiated by EC Casp9 points out key cytokines that could contribute to the development of retinal edema and neurodegeneration, which could be targeted therapeutically. However, one of the limitations of the study is the potential inflammation that could be caused by laser damage. Although it has been reported that sham-lasered exposed retinas expressed an increase in genes or mRNA levels associated with inflammation. The methodology of these studies used a much higher laser exposure time (2.5 s), laser applications (up to six) [68] or a much higher laser power (300 mW) [69]. Some of the cytokines that have been shown to be elevated in sham-lasered eyes are MCP-1, IL-6, and ICAM-1, but this increase has been reported to be present only at 0.5 days P-RVO and measured by mRNA levels [69]. We focused on cytokine protein levels at one day P-RVO and minimized laser-induced injury by decreasing laser exposure, power, and application. It is important to mention as well that RVO pathology is driven by retinal edema which we and others have reported previously not to be present in sham-lasered retinas [10, 69].

While caspase-9 has not been shown to directly activate cytokines, downstream caspase-9 targets such as caspase-6, caspase-7 and caspase-3 are known to cleave cytokine proforms and increase overall cytokine levels [48]. Many of the EC Casp9-regulated cytokines are target genes of the transcription factor NFκ-B (https://www.bu.edu/nf-kb/gene-resources/target-genes/). A recent in vitro enzymatic study suggested that NFκ-B is a potential substrate of caspase-6 [70]. These data suggest that the EC Casp9 regulation of caspase-6 could lead to NFκ-B activation and subsequent mediation of pro-inflammatory cytokines in astrocytes – where we found the most expression of cl-caspase-6 P-RVO.

Most of the cytokines are expressed in microglia, and in RVO a recent study suggested that microglia are a main source of cytokines [8]. In a model of branch RVO (occlusion of one retinal vein) microglia respond by clustering and increasing in number near the site of injury three days P-RVO, with a peak in microglial number at seven days [7]. Our study revealed that RVO leads to an increase in microglial number as early as two-days P-RVO independent of EC Casp9. To better visualize and appreciate individual microglial cells and their localization in retinal layers, we used retinal sections for microglial analysis. One drawback of this technique is that it does not allow for analysis of microglial clusters and mobilization towards the occluded veins. The identification of pro-inflammatory microglia has been ascribed to morphological changes from ramified to ameboid in the context of disease or injury [49]. We found that RVO changes the morphology of microglia towards a bigger, more circular, and hypertrophic shape. This is the first study to quantify RVO-induced microglial morphological changes. However, EC Casp9 regulation of microglial morphology was transient as it was only present at one day P-RVO. It is important to note that a limitation of the microglial morphological analysis used in this study is that it did not target all microglial cells or processes, but this was consistent in all the images that were evaluated.

We also found that Astro Casp9 deletion led to a significant decrease in astroglial caspase-6, suggesting that activation of astroglial caspase-6 occurs in a cell-intrinsic manner. It is important to mention that the levels of astroglial caspase-6 in the iAstro Casp9 KO were not entirely eliminated. This could indicate that some activation of astroglial caspase-6 is due to EC Casp9-astroglial signaling or that the promoter used to generate our mouse model (GCE) does not target all astrocytes in the retina. It is known that GFAP is not expressed in all astrocytes and not all cells that express GFAP are astrocytes [71]. In case of injury, Müller glia can also express GFAP. Therefore, consideration should be taken regarding the conclusions stated if the promoter is not totally specific for retinal astrocytes, the roles for Astro Casp9 in RVO could include a wide range of roles beyond of what we found in this study.

Our results suggest that Astro Casp9 loss did not ameliorate retinal swelling, indicating that Astro Casp9 is not an active participant in the development of retinal edema. However, Astro Casp9 loss protected the retina from capillary ischemia as measured by DRIL. DRIL was characterized by Sun and others as a predictor of decline in visual acuity [72] and capillary nonperfusion [73]. These data indicate that downstream Astro Casp9 signaling could interfere with vascular blood flow and that the contribution of Astro Casp9 to RVO pathology is associated with capillary ischemia. A potential mechanism could be through decreased levels of tight junctions, enzymes, and neurovascular coupling all of which disturb the BRB and lead to ischemia. Lastly, we found that at the acute stage of RVO, Astro Casp9 did not protect neurons from neuronal death. This result sheds light on the contribution of Astro Casp9 signaling and potentially, the cytokines that could be processed by the downstream targets of Astro Casp9 and 6. Moreover, it suggests that capillary ischemia is not a main driver of neuronal death nor retinal edema but is driving contrast sensitivity decline.

We demonstrate an important role for EC Casp9 and Astro Casp9 in RVO-induced inflammation, visual dysfunction, and capillary ischemia (Fig. S6). These findings put into perspective the need to understand cell-specific roles in neurovascular injury and which neuroimmune responses are responsible for retinal edema and visual dysfunction. Further studies targeting endothelial and Astro Casp9 could lead to the development of more potent therapeutics to preserve vision.

## Supplementary information

Supplementary Material

Western blots

Checklist

## Data Availability

The data that support the findings of this study are available from the corresponding author upon reasonable request.
