# Correction: Neurovascular injury associated non-apoptotic endothelial caspase-9 and astroglial caspase-9 mediate inflammation and contrast sensitivity decline

**DOI:** 10.1038/s41419-022-05440-1

**Published:** 2022-11-24

**Authors:** Crystal Colón Ortiz, Albertine M. Neal, Maria I. Avrutsky, Monica Choi, Jade Smart, Jacqueline Lawson, Carol M. Troy

**Affiliations:** 1 Department of Pathology & Cell Biology; Vagelos College of Physicians and Surgeons, Columbia University, New York, NY 10032 USA; 2 Barnard College, Columbia University, New York, NY 10032 USA; 3 Department of Neurology; Vagelos College of Physicians and Surgeons, Columbia University, New York, NY 10032 USA; 4 The Taub Institute for Research on Alzheimer’s Disease and the Aging Brain; Vagelos College of Physicians and Surgeons, Columbia University, New York, NY 10032 USA

**Keywords:** Neuro-vascular interactions, Cell signalling

Correction to: *Cell Death and Disease* 10.1038/s41419-022-05387-3, published online 08 November 2022

The original version of this article contained an error. The following sections were omitted from the print version of the accepted paper: ACKNOWLEDGEMENTS: We would like to thank Natasha Snider for kindly providing the GFAP GA5 antibody along with guidance and recommendations. We would also like to thank Marc Tessier-Lavigne and Ralf Adams for providing the caspase-9 flox/flox and the Cdh5(PAC)-CreERT2 mice, respectively. We would like to thank James Goldman for helpful discussions and suggestions.

FUNDING: The research presented in this publication was supported by the National Science Foundation Graduate Research Fellowship Program (NSF-GRFP) grant DGE – 1644869, the National Institute of Neurological Disorders and Stroke (NINDS) of the National Institutes of Health (NIH), award number F99NS124180 NIH NINDS Diversity Specialized F99 (to CKCO), award number R03NS099920 (to CMT), and the Department of Defense Army/Air Force (DURIP to CMT). The content is the responsibility of the authors and does not represent the views of the National Institutes of Health.

The original article has been corrected.

