# Supplementary material for: Neurovascular injury associated non-apoptotic endothelial caspase-9 and astroglial caspase-9 mediate inflammation and contrast sensitivity decline
Source: Cell Death Dis. 2022 Nov 8;13(11):937. doi: 10.1038/s41419-022-05387-3 (PMC9643361; doi:10.1038/s41419-022-05387-3)
Supplement: Supplementary file 1 — Supplementary Material [file 41419_2022_5387_MOESM1_ESM.docx]

**Cell specific regulation of neurovascular disease by endothelial and astroglial caspase-9**

**C.K. Colón Ortiz et al.**

Supplementary Figures and Information

**
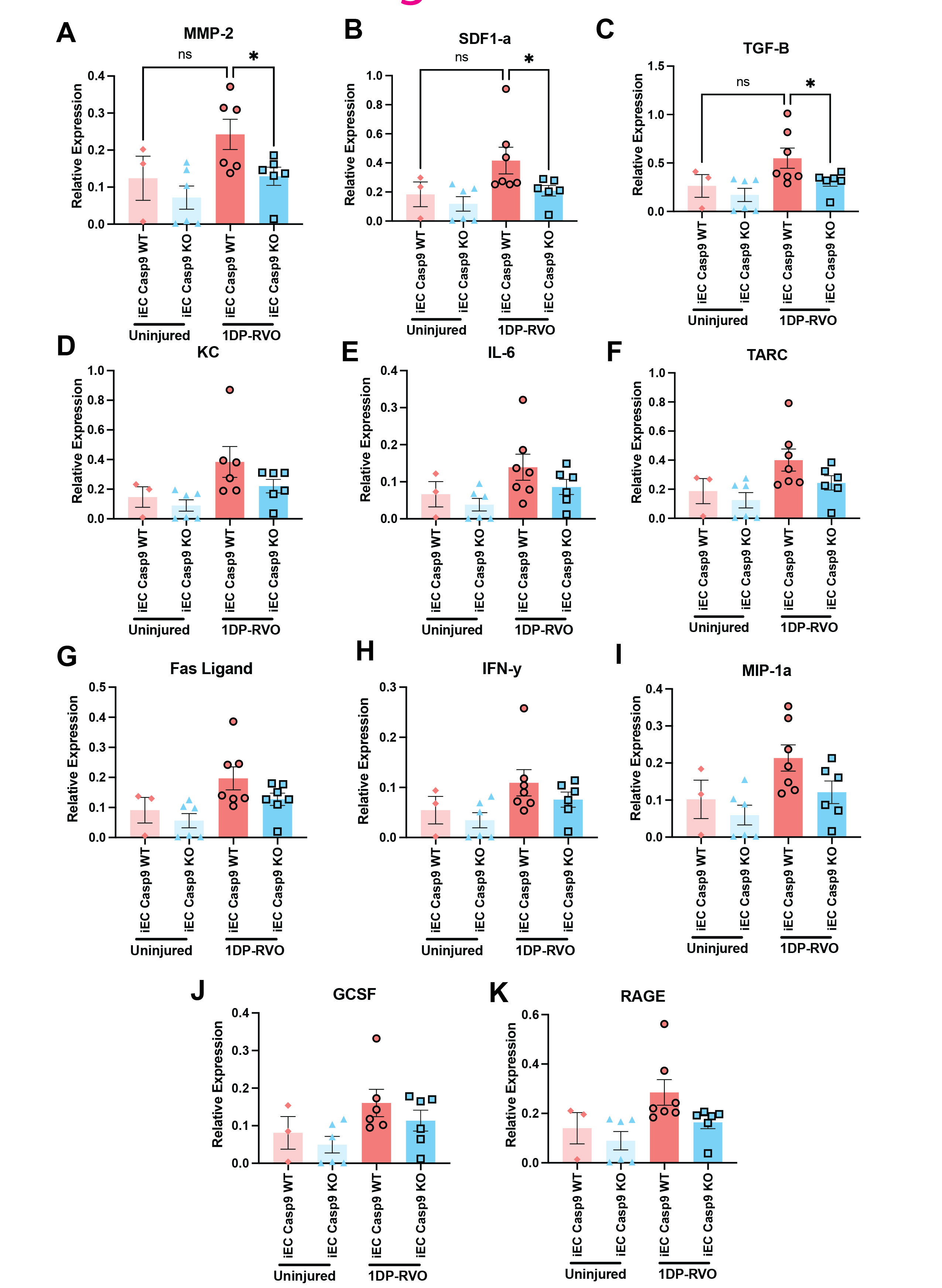
**

**Supplementary Figure 1. Cytokines not modulated by RVO. (A-K)** Relative expression of cytokines in retinas from iEC Casp9 WT in uninjured iEC Casp9 WT (n=3) and KO (n=6) and iEC Casp9 WT (n=6) and KO (n=6) one day P-RVO. Error bars mean ± SEM; One-way ANOVA, Fisher’s LSD test. ******P* ≤0.05.

**
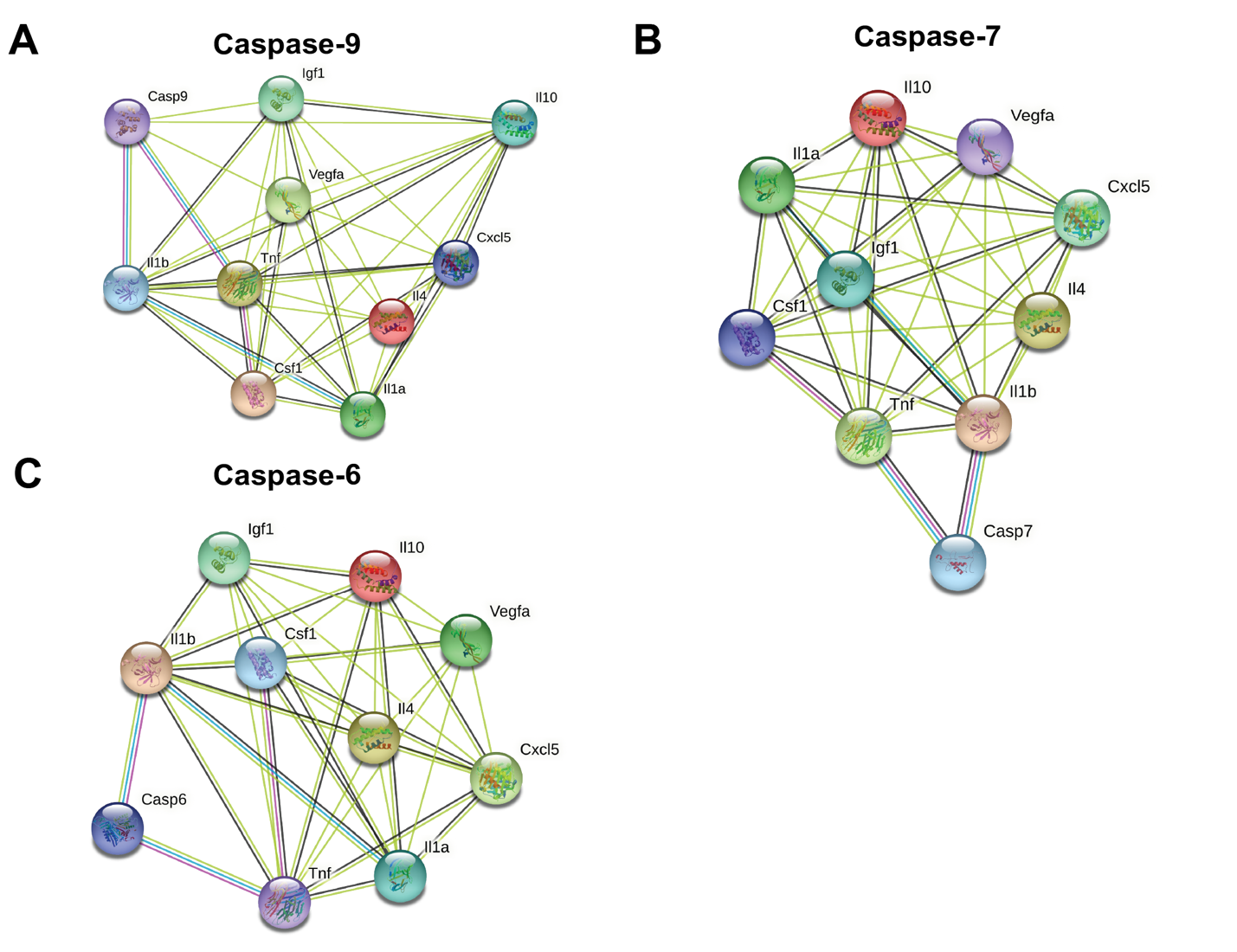
**

**Supplementary Figure 2. STRING analysis of cytokines modified by EC Casp9 deletion. A)** Caspase-9 protein-protein network interaction of cytokines regulated by EC Casp9 P-RVO and downstream caspases **B)** caspase-7 and **C)** caspase-6. Protein-protein interactions; curated databases (aqua), experimentally determined (pink), textmining (light green), and co-expression (black).


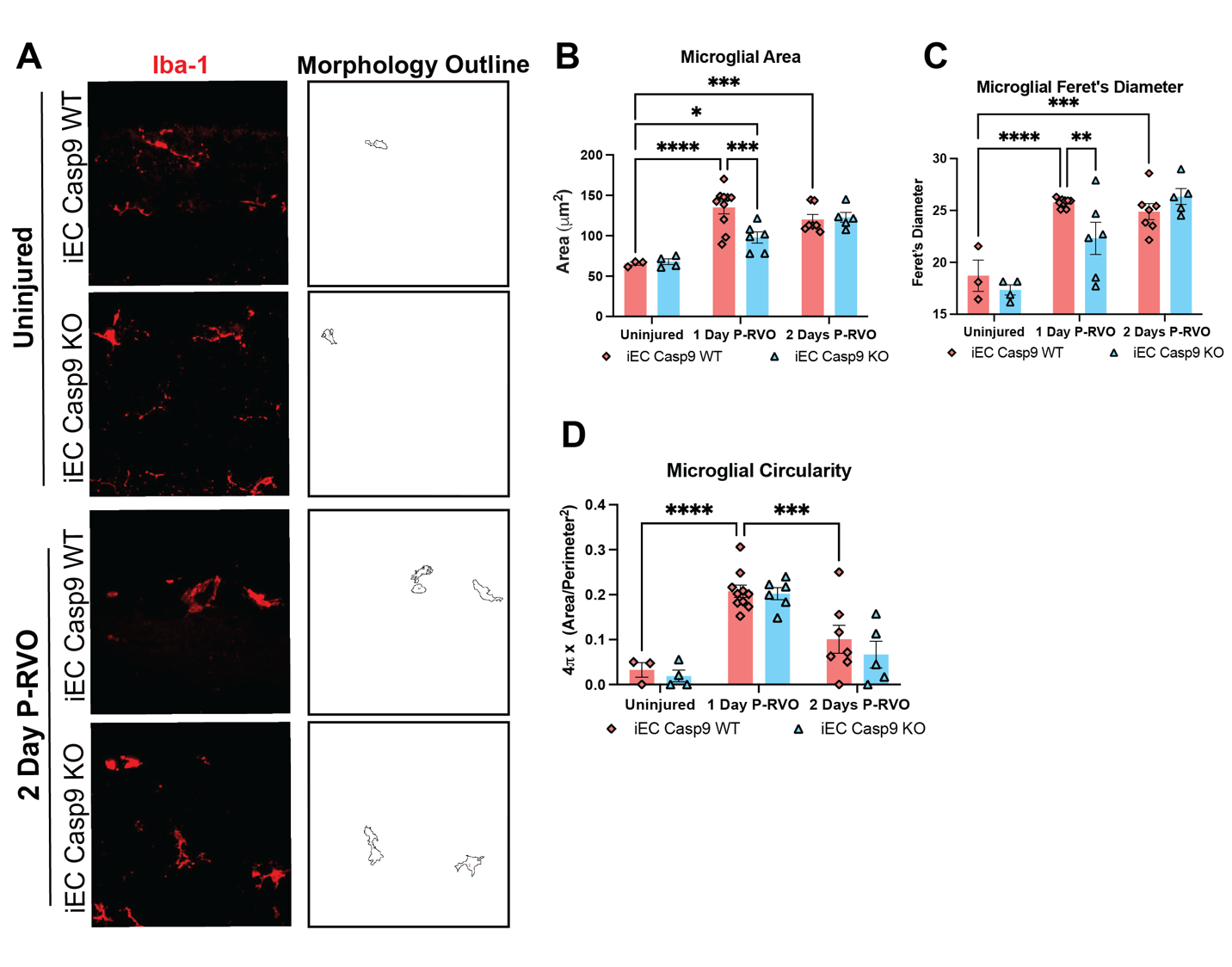


**Supplementary Figure 3. RVO induced microglial morphology changes. A)** Retinal cross-sections from uninjured and two-days P-RVO iEC Casp9 WT and KO mice stained with Iba-1 (red) and morphology outline (white). **B)** Microglial area, **C)** Ferret’s diameter, and **D)** circularity of uninjured iEC Casp9 WT (n=5) and KO (n=6), iEC Casp9 WT (n=8) and KO (n=9) one day P-RVO, and iEC Casp9 WT (n=7) and KO (n=9) two days P-RVO. ******P* ≤0.05, *******P* ≤0.01, ********P* ≤0.001 and *********P* ≤0.0001.


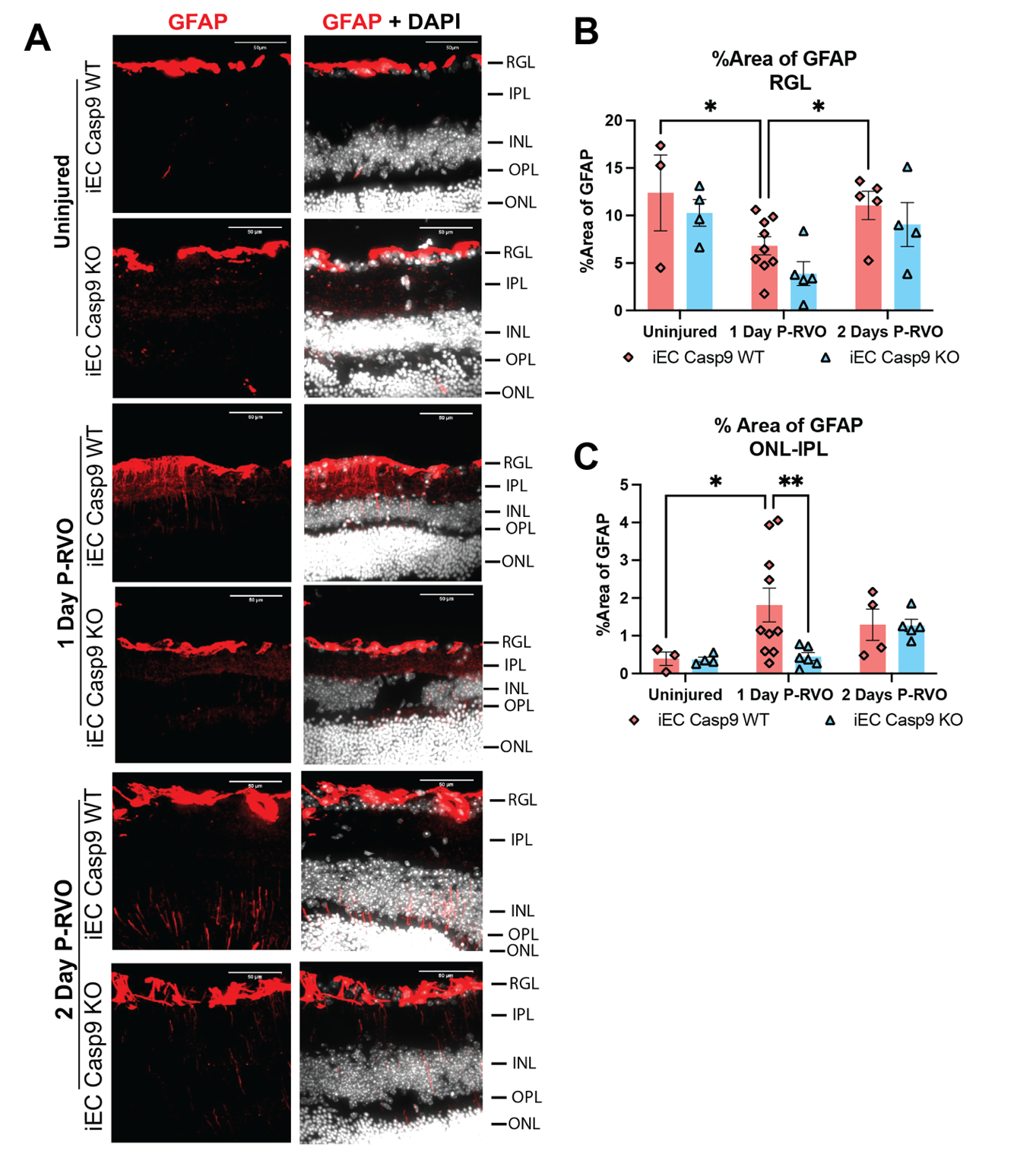
**Supplementary Figure 4. EC Casp9 loss does not change GFAP in macroglia. A)** Retinal cross-sections from uninjured, one and two-days P-RVO iEC Casp9 WT and KO mice stained with GFAP (red) and DAPI (white). Scale bar=50µm. **B)** Quantification of percent area of GFAP expression in RGL. **C)** Quantification of percent area of GFAP expression from ONL-OPL. Both quantifications were done in uninjured iEC Casp9 WT (n= 3) and KO (n= 4) one day P-RVO iEC Casp9 WT (n= 10) and KO (n= 6), and two days P-RVO iEC Casp9 WT (n= 4-5) and KO (n= 4-5). Error bars mean ± SEM; Two-way ANOVA, Fisher’s LSD test. ******P* ≤0.05 and *******P* ≤0.01. Retinal ganglion layer (RGL), inner plexiform layer (IPL), inner nuclear layer (INL), outer plexiform layer (OPL), and outer nuclear layer (ONL).

**
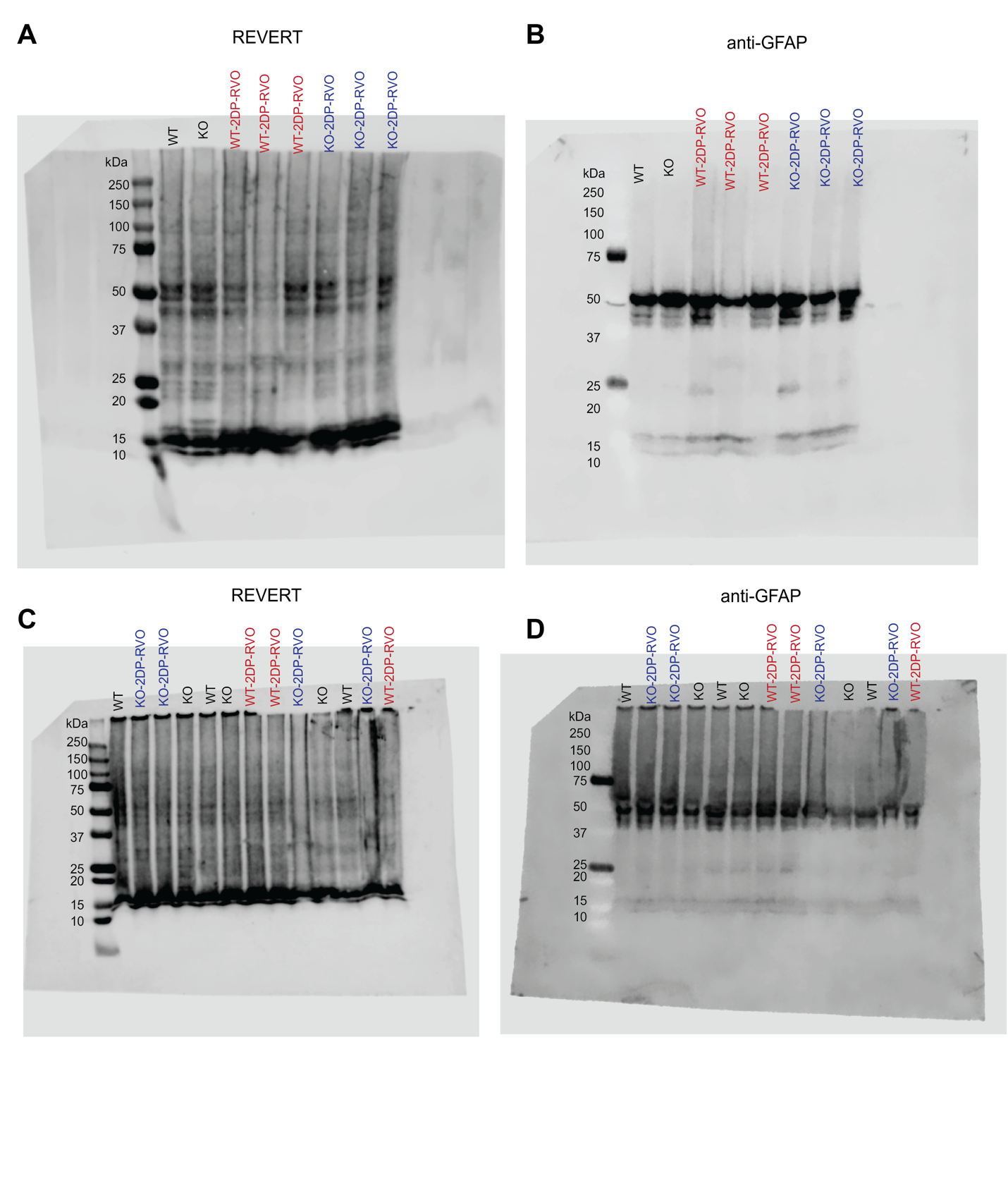
**

**Supplementary Figure 5. EC Casp9 mediates caspase-6 cl-GFAP.** **A)** REVERT total protein stain blot of uninjured and injured (2DP-RVO) iEC Casp9 WT/KO retinal lysates. **B)** Western Blot stained for GFAP with GFAP GA5 Sigma 63893. **C)** REVERT total protein stain blot of biological replicates uninjured and injured (2DP-RVO) iEC Casp9 WT/KO retinal lysates. **D)** Western Blot of biological replicates stained for GFAP with GFAP GA5 Sigma 63893.


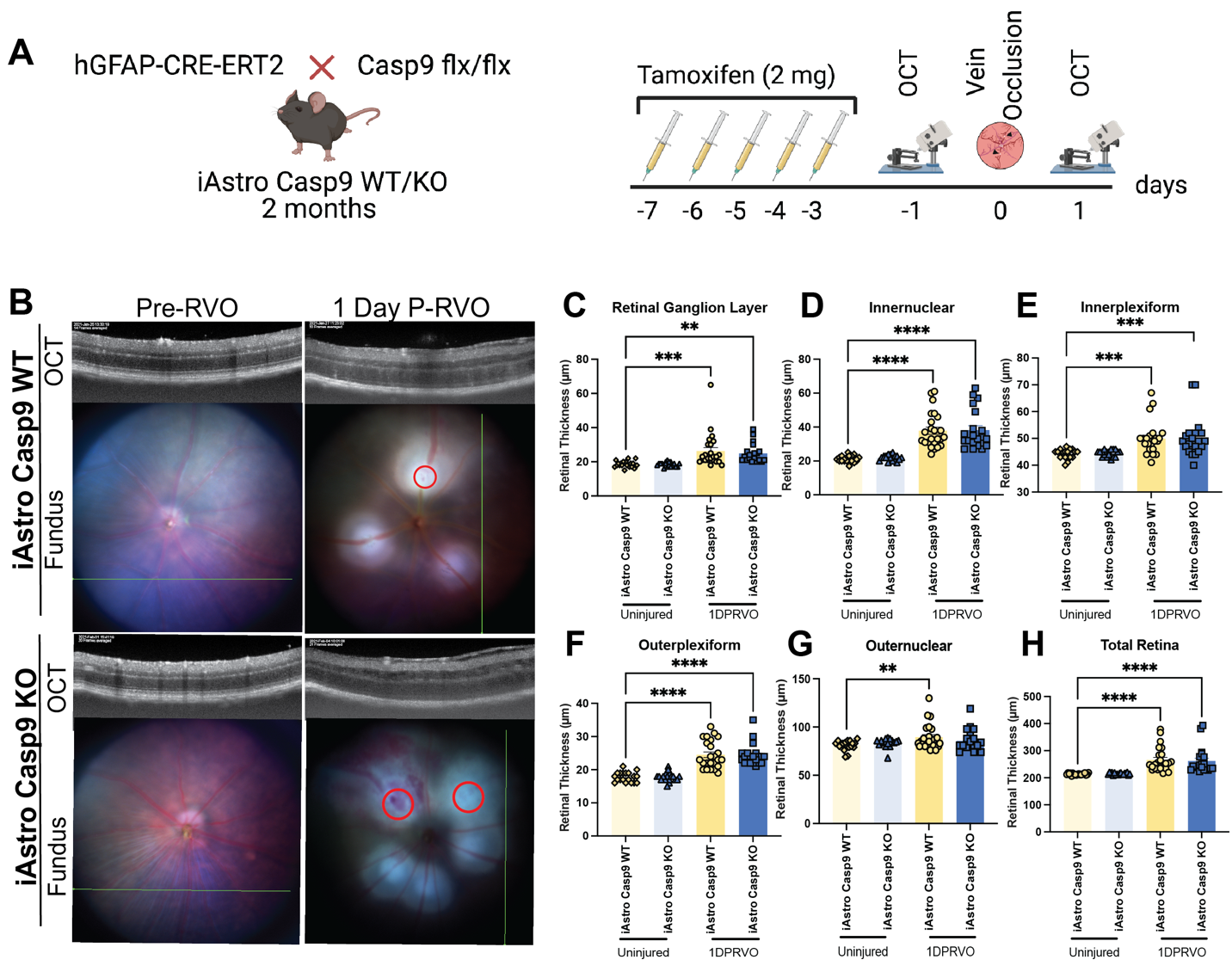
**Supplementary Figure 6. Astro Casp9 deletion does not protect from retinal edema.** **A)** Experimental schematic. Two-month-old iAstro Casp9 WT/KO mice were treated with tamoxifen for five consecutive days. After two days, animals were subjected to RVO, and retinal thickness was measured pre-RVO and one day P-RVO. Created with BioRender.com. **B)** Representative retinal fundus and OCT pre-RVO and one day P-RVO of iAstro Casp9 WT and KO. **C-H)** Thickness of retinal layers of uninjured and one day P-RVO iAstro Casp9 WT and KO. *******P* ≤0.01, ********P* ≤0.001 and *********P* ≤0.0001. Error bars mean ± SEM; One-way ANOVA, Fisher’s LSD test.

**
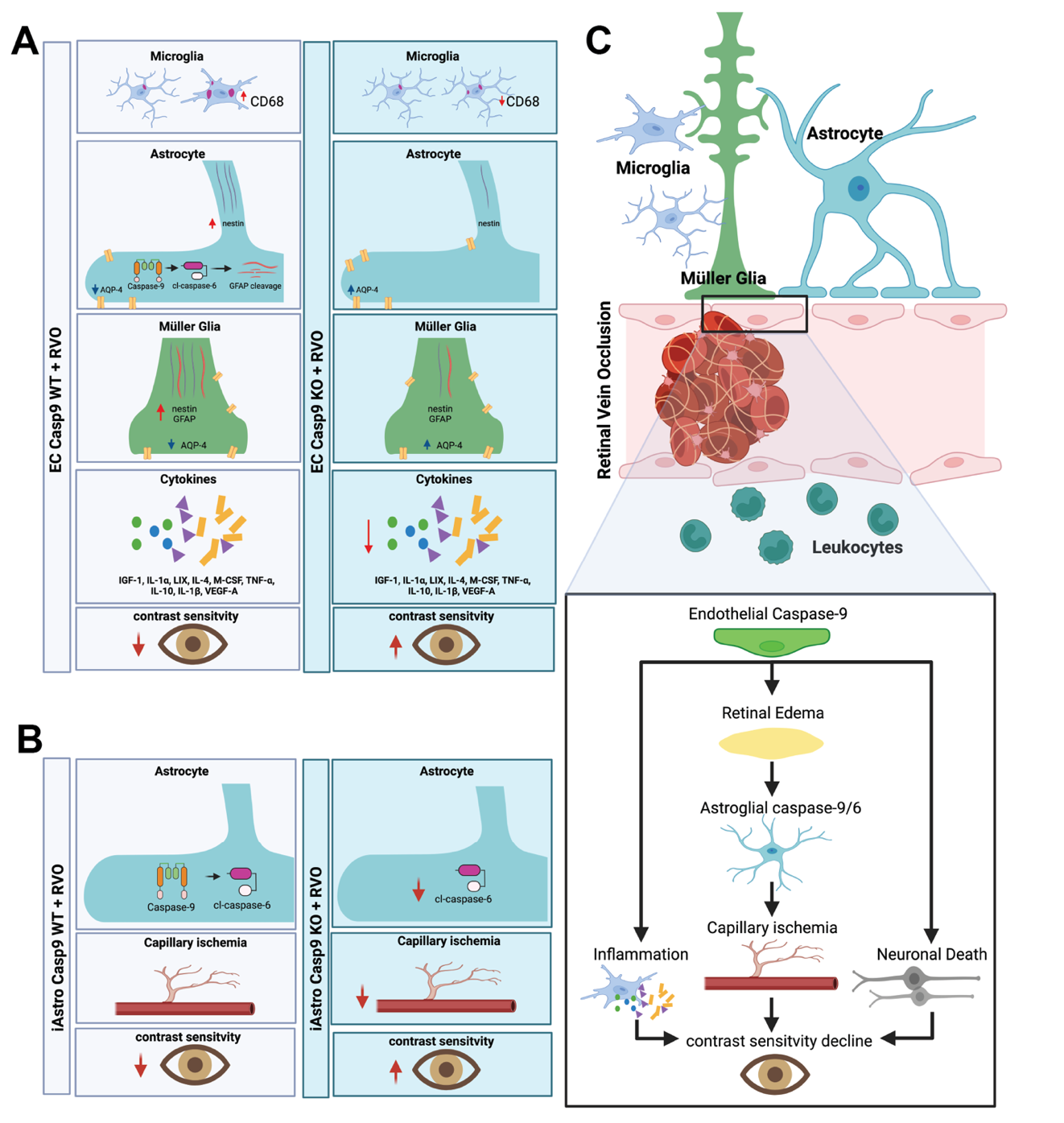
**

**Supplementary Figure 7. Endothelial and Astro Casp9 mediate inflammation and contrast sensitivity decline. A)** Summarized observations of deletion of EC Casp9 on glial cells and contrast sensitivity response P-RVO. **B)** Summarized observations of deletion of Astro Casp9 on astroglial cl-caspase-6, capillary ischemia, and contrast sensitivity response P-RVO. **C)** Proposed pathway based on previous (Avrutsky et al., 2020) and presented data. Neurovascular injury leads to non-apoptotic activation of EC Casp9 which causes increase on retinal edema, neuronal death, inflammation, and activation of Astro Casp9/6. Astro Casp9 then contributes to capillary ischemia and in conjunction with EC Casp9, contrast sensitivity decline.
