# Supplementary material for: Neurovascular injury associated non-apoptotic endothelial caspase-9 and astroglial caspase-9 mediate inflammation and contrast sensitivity decline
Source: Cell Death Dis. 2022 Nov 8;13(11):937. doi: 10.1038/s41419-022-05387-3 (PMC9643361; doi:10.1038/s41419-022-05387-3)
Supplement: Supplementary file 2 — Western blots [file 41419_2022_5387_MOESM2_ESM.docx]

**
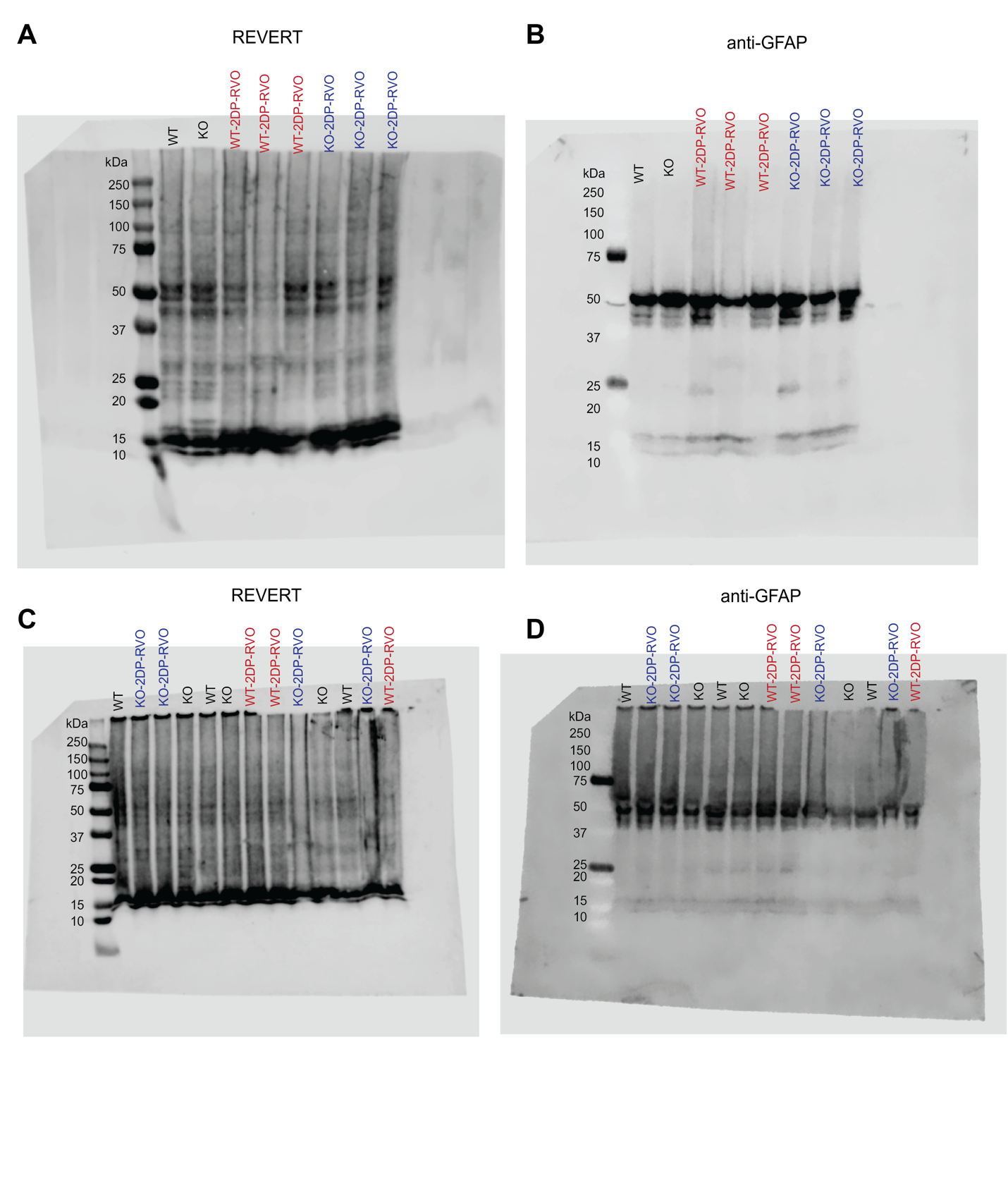
**

**Full Western blots for Figure 5. EC Casp9 mediates caspase-6 cl-GFAP.** **A)** REVERT total protein stain blot of uninjured and injured (2DP-RVO) iEC Casp9 WT/KO retinal lysates. **B)** Western Blot stained for GFAP with GFAP GA5 Sigma 63893. **C)** REVERT total protein stain blot of biological replicates uninjured and injured (2DP-RVO) iEC Casp9 WT/KO retinal lysates. **D)** Western Blot of biological replicates stained for GFAP with GFAP GA5 Sigma 63893.
